# CT-Based Radiomics Showing Generalization to Predict Tumor Regression Grade for Advanced Gastric Cancer Treated With Neoadjuvant Chemotherapy

**DOI:** 10.3389/fonc.2022.758863

**Published:** 2022-02-25

**Authors:** Yong Chen, Wei Xu, Yan-Ling Li, Wentao Liu, Birendra Kumar Sah, Lan Wang, Zhihan Xu, Michael Wels, Yanan Zheng, Min Yan, Huan Zhang, Qianchen Ma, Zhenggang Zhu, Chen Li

**Affiliations:** ^1^ Department of Radiology, Ruijin Hospital, Shanghai Jiao Tong University School of Medicine, Shanghai, China; ^2^ Department of General Surgery, Shanghai Key Laboratory of Gastric Neoplasms, Shanghai Institute of Digestive Surgery, Ruijin Hospital, Shanghai Jiao Tong University School of Medicine, Shanghai, China; ^3^ Key Laboratory of Carcinogenesis and Translational Research (Ministry of Education/Beijing), Department of Radiology, Peking University Cancer Hospital and Institute, Beijing, China; ^4^ Siemens Healthineers Ltd., Shanghai, China; ^5^ Department of Diagnostic Imaging Computed Tomography Image Analytics, Siemens Healthcare GmbH, Forchheim, Germany; ^6^ Department of Pathology, Ruijin Hospital, Shanghai Jiao Tong University School of Medicine, Shanghai, China

**Keywords:** radiomics, gastric cancer, neoadjuvant therapy, tumor regression grade, generalization

## Abstract

**Objective:**

The aim of this study was to develop and validate a radiomics model to predict treatment response in patients with advanced gastric cancer (AGC) sensitive to neoadjuvant therapies and verify its generalization among different regimens, including neoadjuvant chemotherapy (NAC) and molecular targeted therapy.

**Materials and Methods:**

A total of 373 patients with AGC receiving neoadjuvant therapies were enrolled from five cohorts. Four cohorts of patients received different regimens of NAC, including three retrospective cohorts (training cohort and internal and external validation cohorts) and a prospective Dragon III cohort (NCT03636893). Another prospective SOXA (apatinib in combination with S-1 and oxaliplatin) cohort received neoadjuvant molecular targeted therapy (ChiCTR-OPC-16010061). All patients underwent computed tomography before treatment, and thereafter, tumor regression grade (TRG) was assessed. The primary tumor was delineated, and 2,452 radiomics features were extracted for each patient. Mutual information and random forest were used for dimensionality reduction and modeling. The performance of the radiomics model to predict TRG under different neoadjuvant therapies was evaluated.

**Results:**

There were 28 radiomics features selected. The radiomics model showed generalization to predict TRG for AGC patients across different NAC regimens, with areas under the curve (AUCs) (95% interval confidence) of 0.82 (0.76~0.90), 0.77 (0.63~0.91), 0.78 (0.66~0.89), and 0.72 (0.66~0.89) in the four cohorts, with no statistical difference observed (all p > 0.05). However, the radiomics model showed poor predictive value on the SOXA cohort [AUC, 0.50 (0.27~0.73)], which was significantly worse than that in the training cohort (p = 0.010).

**Conclusion:**

Radiomics is generalizable to predict TRG for AGC patients receiving NAC treatments, which is beneficial to transform appropriate treatment, especially for those insensitive to NAC.

## Introduction

Gastric cancer (GC) is a serious health problem in the world, causing an estimated 783,000 deaths in 2018 (1). Despite surgery being the only curative approach, more than half of cases are initially diagnosed as advanced disease, with a limited 5-year survival of 20%–30% (2). Meanwhile, even after R0 gastrectomy, relapse rates remain high, in the range of 40%–60% (2).

Neoadjuvant chemotherapy (NAC) is beneficial to improving R0 resection and prognosis in patients with advanced gastric cancer (AGC) by downstaging the tumor, eradicating micrometastasis, and reducing the risk of recurrence (3). Compared with only about half of patients with a good condition suitable to receive postoperative chemotherapy, NAC (the preoperative part of perioperative chemotherapy) is feasible to most patients, highlighting its importance (4). Although accumulated studies have been made to investigate regimens with more safety and effectiveness since the landmark MAGIC study launched in 2006, a considerable proportion of cases are insensitive to NAC, leaving unnecessary cytotoxicity to those patients (4–9). Even for the newly reported triplet FLOT regimen (docetaxel, oxaliplatin, fluorouracil, and leucovorin), which is under impassioned discussion as the new standard for NAC, completed or subtotal pathological regression was achieved in only 37% of cases (6). Uniform standard of care is an absence in NAC even if great progress has been achieved in this area (10, 11). Besides, the emergence of treatments for precision medicine, such as molecular targeted therapy and immunotherapy, brings vitality to neoadjuvant approaches (12). The choice of appropriate treatment is beset with difficulties where controversial results were observed from different studies (12). It is of urgent need to find an easy-to-use and noninvasive tool to predict tumor sensitivity to different neoadjuvant regimens.

Transformation in artificial intelligence (AI) has provoked a new area of medical image analysis named radiomics, which noninvasively provides insight into tumor heterogeneity by extracting and analyzing high-throughput image features (13, 14). Radiomics extends the scope of biopsy, providing possibilities in dynamic surveillance, prognosis prediction, and treatment decision (15). Especially, compared with conventional imaging metrics, radiomics effectively gauges tumor microenvironment status, providing tailored treatment for individuals (16–18). Radiomics also allows for predicting treatment sensitivity to non–small cell lung cancer treated with different systemic anticancer therapies (19). Accumulated evidence has identified the prognostic value of radiomics in evaluating tumor sensitivity for GC, but there is a lack of clear elucidation whether radiomics is generalizable among different anticancer regimens (20–22).

In this study, we aimed to predict treatment response for GC patients sensitive to neoadjuvant therapies using radiomics and verify the generalization of this AI technology among different regimens, including NAC regimens and molecular targeted therapy.

## Materials and Methods

The main objective was to test whether the radiomics model we built was generalizable to detect AGC tumors sensitive to neoadjuvant approaches. We firstly trained and tested the radiomics model on baseline CT images for patients treated with the EOX regimen (epirubicin, oxaliplatin, and capecitabine). Then, we sought to investigate whether this model was practical on real-world data by applying it to predict therapeutic response on a clinical trial [the Dragon III study (23), ClinicalTrials.gov: NCT03636893] and a retrospective external validation cohort incorporated with three mixture NAC regimens. We finally aimed to validate whether our radiomics model could predict treatment response to molecular targeted therapy from another clinical trial (ChiCTR.gov.cn: ChiCTR-OPC-16010061) (24).

### Participants

**Figure 1:**
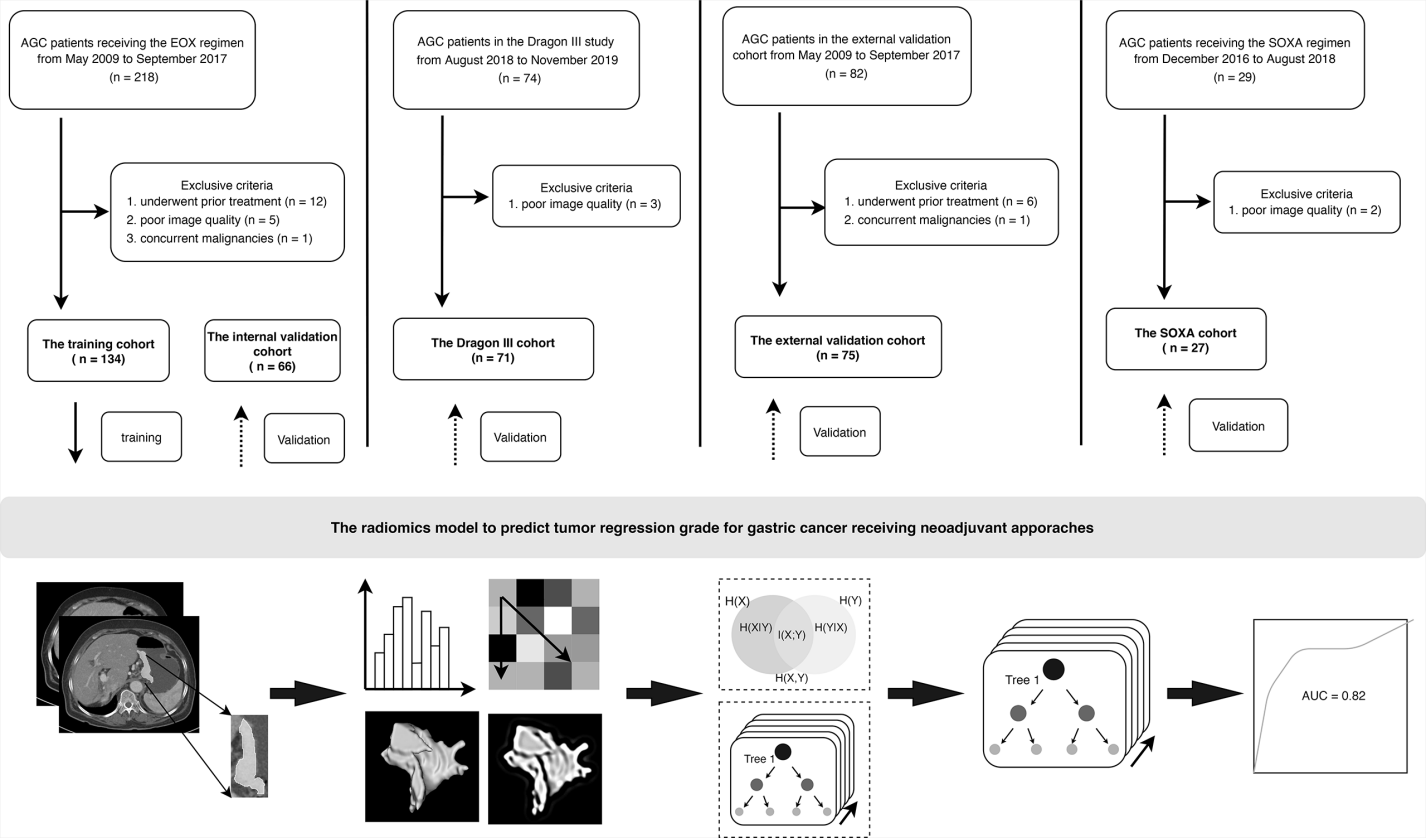
Flowchart and patient enrollment of this study. AGC, advanced gastric cancer. For the regimens, EOX (epirubicin, oxaliplatin, and capecitabine), SOXA (apatinib in combination with S-1 and oxaliplatin).

Patients consecutively enrolled in this study were histopathologically diagnosed as having gastric adenocarcinoma and with advanced disease (cT2-4a/bNxM0) based on contrast-enhanced CT (CECT) images. No prior treatment before NAC was demanded, and all patients underwent CECT scans less than 3 weeks before treatment started. Meanwhile, all patients enrolled were tolerant of NAC and have completed the planned preoperative schedule. The enrollment for all patients is presented in **Figure 1**.

Patients receiving the triplet EOX regimen were retrospectively recruited from May 2009 to September 2017. A total of 200 patients (women, 64; mean age, 59.5 ± 9.7 years) were finally recruited and were divided into a training cohort and an internal validation cohort to construct the radiomics model at a ratio of 2:1. Therefore, there were 134 and 66 patients in the training and internal validation cohorts, respectively.

The Dragon III study was sought to compare the efficacy between the doublet SOX regimen (S-1 and oxaliplatin) and the triplet FLOT regimen. This clinical trial was conducted from August 2018 to November 2019. Finally, there were 71 patients (women, 24; mean age, 61.3 ± 9.9 years) enrolled in this study, and three patients were excluded due to poor image quality of CECT. Those patients receiving the two regimens were considered as an entity and named as the Dragon III cohort. There were 27 and 44 patients receiving the SOX regimen and the FLOT regimen in the Dragon III cohort, respectively.

Patients in the external validation cohort were from another tertiary referral hospital (Peking University Cancer Hospital and Institute) and were retrospectively recruited from January 2018 to December 2019. In this cohort, three doublet NAC regimens were used, including the SOX regimen, the XELOX regimen (oxaliplatin and capecitabine), and the FOLFOX regimen (oxaliplatin, folinic acid, and fluorouracil). There were 75 AGC patients (women, 24; mean age, 59.4 ± 10.5 years) enrolled in this cohort. There were 42, 30, and 3 patients receiving the SOX, the XELOX, and the FOLFOX regimens, respectively.

Patients in the clinical trial (ChiCTR.gov.cn: ChiCTR-OPC-16010061) were recruited from December 2016 to August 2018. The single-arm, open-label, phase II trial was designed to investigate the added value of apatinib in combination with SOX (the SOXA regimen) to improve the pathologic response of AGC patients. Two patients were excluded due to poor image quality of CECT, and 27 patients (women, 10; mean age, 59.3 ± 8.0 years) were enrolled in this study as the SOXA cohort.

This study was approved by the ethics committee of Ruijin Hospital and Peking University Cancer Hospital and Institute. For patients in the training, internal validation, and external validation cohorts, written informed consent was waived due to the retrospective nature. Baseline clinical data for all patients included gender, age, tumor invasion, lymph node status, tumor location, tumor size (cm), differentiation status, carcinoembryonic antigen (CEA), and carbohydrate antigen 19-9 (CA19-9). Definition for those indices is presented in the **Supplementary Material S1**.

### Protocols for Neoadjuvant Regimens

For patients receiving the SOX and FLOT regimens in the DRAGON III study (i.e., the DRAGON III cohort) and patients receiving the SOXA regimen (the SOXA cohort), the protocols are described in the corresponding clinical trials (23, 24). Treatment protocols for patients receiving the EOX regimen and patients in the external validation cohort are presented in **Supplementary Material S2**.

### Imaging Protocol

All patients underwent abdomen CECT scanning before treatment. The information and parameters for involved CT scanners are presented in **Supplementary Table S1**. The images on portal-venous and delayed phases were anonymously retrieved for further analysis.

### Tumor Regression Grade

Evaluation of tumor response to treatment was in accordance with Ryan criteria (25) after the completion of planned neoadjuvant courses, where completed response represents no viable cancer cells, moderate response represents single cells or small groups of cancer cells, minimal response represents residual cancer outgrown by fibrosis, and poor response represents minimal or no tumor kill (extensive residual cancer). Two pathologists, who had 7 and 10 years of experience, respectively, evaluated tumor response and resolved disputes with consensus. In this study, we considered patients with a completed response or moderate response to neoadjuvant treatment as responders and minimal response or poor response as non-responders.

### Tumor Delineation and Feature Extraction

Before segmentation, all images were resampled into a uniform voxel space of 1 * 1 * 1 mm. The volume of interest (VOI) was performed on the primary tumor by open-source software (3d-slicer, version 4.10.2) on baseline axial images from portal-venous and delayed phases along with tumor boundary slice by slice and omitting the first and last slices to avoid potential partial volume effect. Radiomics features were extracted from an in-house research platform (Syngo Via, Version VB20, Research Frontier, Siemens Healthineer) (26).

A total of 110 original radiomics features (without any preprocessing producers applied) were extracted from each phase. In addition, 1,116 features were calculated after preprocessing for each phase. Therefore, there were 2,452 radiomics features finally extracted for each patient. All features were compliant with the benchmarks of the image biomarker standardization initiative (IBSI) (27). Detailed information is presented in **Supplementary Material S3**.

To guarantee reproducibility of the radiomics features, we performed reliability analysis by repeatedly delineating the tumors by two radiologists (with 10 years and 5 years of experience in abdominal imaging, respectively) on images from portal-venous and delayed phases from 70 randomly selected patients from the training cohort. The two radiologists were aware of GC but unaware of pathological and clinical results. Intraclass correlation coefficient (ICC) analysis was used for reliability analysis, and features with ICC >0.80 were considered robust and remained for further analysis.

### Dimensionality Reduction, Radiomics Score Generation, and Modeling

To avoid dimensionality curse and overfitting, mutual information that measures the mutual dependence between features and label was used to select candidate features in the training cohort. Random forest (RF), a widely used ensemble machine learning algorithm, was chosen in our studies for radiomics score generation and model establishment (28,29). RF was generally reported to own the best discriminative and diagnostic power in radiomics studies due to their ability to handle high dimensional features and strong generalization. Grid search with 10-fold cross-validation was implemented for parameter tunings in the training cohort. The contribution of features to modeling was ranked by feature importance calculated by Gini-impurity. Subsequently, the radiomics scores that were generated by the established model for every patient were calculated and included for further analysis. The performance of the radiomics model was then verified in the internal validation cohort.

### Generalization Analysis

To investigate the generalization of our radiomics model among different regimens, we further applied the model to predicting TRG for patients in the DRAGON III cohort and the external validation cohort (each included two and three different regimens). Besides, to test whether the model was generalizable to predict treatment response undergoing molecular targeted therapy, we used it to assess TRG on patients in the SOXA cohort.

### Model Comparison

For comparison, we evaluated the degree of tumor response on CT images in accordance with the Response Evaluation Criteria in Solid Tumors (RECIST, version 1.1) (30) for patients receiving the EOX regimen and compared its performance with our radiomics model. In addition, the clinical model was built in the training cohort and validated in corresponding cohorts as the radiomics model did by incorporating significant clinical indices into the multivariate logistic regression model after univariate analysis. Furthermore, we also integrated the radiomics scores and significant clinical characteristics into a combined model to investigate whether there was any improvement to predict TRG for AGC patients in different regimens.

### Subgroup Analysis

Given the SOX regimen and the FLOT regimen indicating no significant TRG difference in the Dragon III study, which was inconsistent with previous studies, we emphasized the comparison between the SOX regimen and the FLOT regimen in the Dragon III cohort. We were also interested in the added value of apatinib in the SOXA cohort compared with patients receiving the SOX regimen only and compared the difference between the two regimens. For patients receiving the SOX regimen, only those recruited from the randomized controlled trial (RCT) clinical trial (i.e., patients in the Dragon III cohort) were considered to match the single-arm SOXA clinical trial. To avoid the influence of response rate within different cohorts, we further split responders and non-responders in each regimen and independently compared them between the regimens. To simplify the analysis, we analyzed the top 10 features in subgroup analysis according to the importance of ranking.

### Statistical Analysis

Continuous variables were compared by independent-samples t-test or Mann–Whitney U test based on their distribution. Categorical variables were compared using χ^2^ or Fisher’s exact test. For model assessment, discrimination ability included receiver operating characteristic (ROC) curve and area under the curve (AUC), sensitivity, specificity, and accuracy were performed for all models. The optimal threshold was selected based on the Youden index for the radiomics score in the training cohort, and Delong test was implemented for the AUC comparison between models in each cohort. The reliability of those models was evaluated by Cohens’ kappa index. An individualized nomogram and a decision curve were implemented to present the clinical utility of our models. The goodness of fit of all models was assessed by calibration curve and Briers score. The radiomics analysis was compliant with published guidance, and we performed a radiomics quality score (RQS) for our study to assess the quality of our study (15). All statistical analyses were performed with software R (version 3.6.0, http://www.r-project.org) and Python Scikit-learn package (version 3.7, Scikit-learn version 0.24.1, https://scikit-learn.org/stable/index.html). Package resources are listed in **Supplementary Table S2**. Statistical significance was considered as a two-sided p < 0.05.

## Results

### Clinical Characteristics

Demographic data in each cohort are presented in **Table 1**. For patients receiving the EOX regimen, there was no response bias between the training cohort and the internal validation cohort (73.1% vs. 75.8%, p = 0.691). Univariate analysis for differentiation status was not performed, given 28% of cases were not accurately evaluated by a specimen from the endoscopic biopsy. For other clinical indices, only age and tumor size were statistically different between responders and non-responders in the training cohort (p = 0.023 and p = 0.020, respectively). Gender, tumor invasion, lymph node status, tumor location, CA19-9, and CEA showed no statistical difference (all p > 0.05, see **Supplementary Table S3**). Finally, we integrated age and tumor size as clinical risk factors into a clinical model using multivariate logistic regression.

**Table 1:** Demographic data for all patients in each cohort.

	The training cohort (n = 134)	The internal validation cohort (n = 66)	The Dragon III cohort (n = 71)	The external validation cohort (n = 75)	The SOXA cohort (n = 27)	p value
**TRG (response, %)**	98 (73.1%)	50 (75.8%)	13 (18.3%)	23 (30.7%)	16 (59.3%)	<0.001
**Regimens**	EOX	EOX	SOX and FLOT	SOX, XELOX and FOLFOX	apatinib plus SOX	
**Age (years)**	58.4 ± 10.2	60.3 ± 8.8	61.3 ± 9.9	59.4 ± 10.5	59.3 ± 8.0	0.328
**Gender (female, %)**	41 (30.6%)	23 (34.8%)	24 (33.8%)	25 (33.3%)	9 (33.3%)	0.977
**Tumor invasion**						<0.001
cT2	0 (0.0%)	0 (0.0%)	0 (0.0%)	6 (8.0%)	0 (0.0%)	
cT3	0 (0.0%)	0 (0.0%)	0 (0.0%)	23 (28%)	0 (0.0%)	
cT4	134 (100.0%)	66 (100%)	71 (100.0%)	46 (61.4%)	27 (100.0%)	
**Lymph node status**						<0.001
cN0	0 (0.0%)	0 (0.0%)	4 (5.6%)	3 (4.0%)	0 (0.0%)	
cN1	23 (17.2%)	5 (7.6%)	10 (14.1%)	26 (34.7%)	7 (25.9%)	
cN2	55 (41.0%)	29 (43.9%)	48 (67.6%)	29 (38.7%)	15 (55.6%)	
cN3	56 (41.8%)	32 (48.5%)	9 (12.7%)	17 (22.7%)	5 (18.5%)	
**Tumor size (cm)**	6.46 ± 2.32	7.16 ± 1.90	5.64 ± 1.96	5.23 ± 1.64	4.83 ± 2.38	<0.001
**Tumor location**						0.002
Upper	33 (24.6%)	16 (24.2%)	23 (32.4%)	18 (24.0%)	14 (51.9%)	
Middle	38 (28.4%)	18 (27.3%)	4 (5.6%)	15 (20.0%)	3 (11.1%)	
Lower	41 (30.6%)	19 (28.8%)	36 (50.7%)	28 (37.3%)	5 (18.5%)	
Diffuse	22 (16.4%)	13 (19.7%)	9 (11.3%)	14 (18.7%)	5 (18.5%)	
**Differentiation status**						<0.001
Well differentiated	2 (1.5%)	0 (0%)	0 (0%)	1 (1.3%)	1 (3.7%)	
Moderately differentiated	11 (8.2%)	4 (6.1%)	9 (12.7%)	41 (54.7%)	3 (11.1%)	
Poorly differentiated	78 (58.2%)	35 (53.0%)	57 (80.3%)	33 (44%)	15 (55.6%)	
SRCC	8 (6.0%)	6 (9.1%)	1 (1.4%)	0 (0%)	1 (3.7%)	
Not evaluated	35 (26.1%)	21 (31.8%)	4 (5.6%)	0 (0%)	7 (25.9%)	
**CA19-9 (positive, %)**	43 (32.1%)	16 (24.2%)	13 (18.3%)	7 (9.3%)	5 (18.5%)	0.005
**CEA (positive, %)**	39 (29.1%)	24 (36.4%)	18 (25.4%)	12 (16.2%)	5 (18.5%)	0.061

CA19-9, carbohydrate antigen 19-9; CEA, carcinoembryonic antigen; SRCC, signet-ring cell carcinoma; TRG, tumor regression grade. For the regimens, EOX (epirubicin, oxaliplatin, and capecitabine), FOLFOX (oxaliplatin, folinic acid and 5-fluorouracil), FLOT (5-fluorouracil, leucovorin, docetaxel and oxaliplatin), SOX (S-1 and oxaliplatin), SOXA (apatinib in combination with SOX), XELOX (oxaliplatin and capecitabine).

### Dimensionality Reduction

The average ICCs for features from the portal-venous and the delayed phase images were 0.75 and 0.80, respectively. There were 668 and 776 radiomics features with a threshold of 0.80 in each phase, and finally, a total of 1,444 radiomics features were considered as robust and remained for further dimensionality reduction.

**Figure 2:**
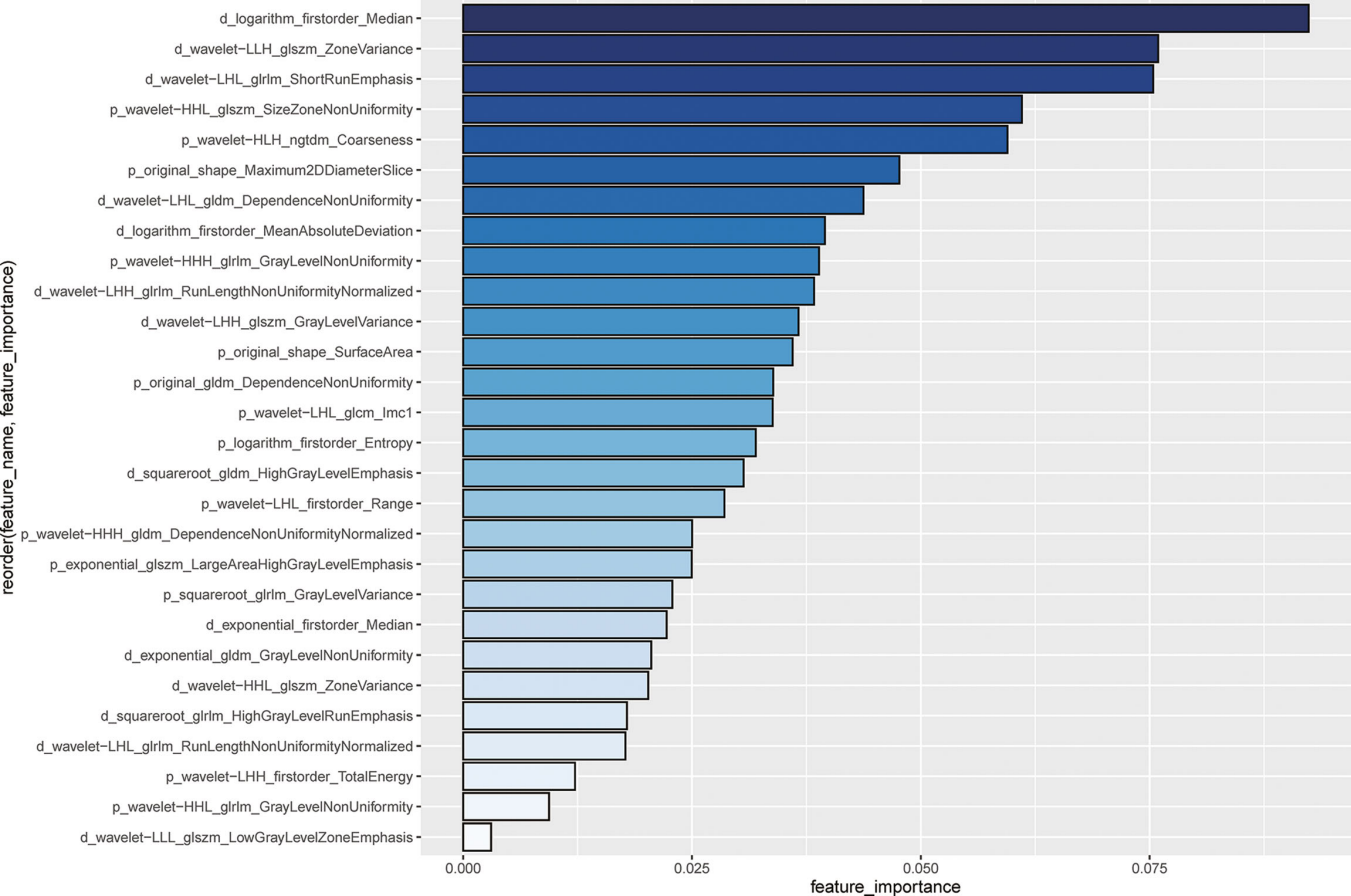
Importance ranking for 28 selected radiomics features using random forest The length of the bin and the depth of the color blue represent the important degree of the radiomics features. The feature name is ordered by the following rule: phase (p or d represents the venous-portal phase or the delayed phase) _ pre-processing_feature category_feature name. For example, for the first feature, i.e., d_logarithm_firstorder_Median indicates that the feature is named as Median from the first-order category, with transformation by logarithm.

A total of 741 radiomics features showed non-zero mutual information. Subsequently, 164 radiomics features were selected with a threshold value of 0.05. Finally, 28 features with the highest importance ranking were used for modeling after RF (**Figure 2**). The RQS of our study was 30 (83.3% of ideal score), guaranteeing good quality of the radiomics process (**Supplementary Table S4**).

### Modeling

We utilized the 28 features to construct a radiomics model to predict TRG for AGC patients. The radiomics score of the training cohort was 0.600. The model achieved AUCs of 0.82 (95% CI, 0.76~0.90) and 0.77 (95% CI, 0.63~0.91) in the training and internal validation cohorts in patients treated with the EOX regimen. Furthermore, the radiomics model showed generalizability to other NAC regimens because similar performance was observed [for the Dragon III cohort and the external validation cohort, AUCs of 0.78 (95% CI, 0.66~0.89) and 0.72 (95% CI, 0.66~0.89), respectively]. Delong test indicated that no significant difference was found when compared with the training cohort (p = 0.571 and p = 0.216, respectively). However, the radiomics model showed a poor predictive value for TRG in the SOXA cohort [AUC of 0.50 (95% CI, 0.27~0.73) vs. 0.82 in the training cohort, p = 0.010]. Detailed information for the performance of the radiomics model in each cohort is presented in **Table 2**. The radiomics score for patients in each cohort is shown in **Supplementary Figure S1**. The Kappa indices of the radiomics models of the training cohort, the internal validation cohort, the Dragon III cohort, the external validation cohort, and the SOXA cohort were 0.485, 0.365, 0.221, 0.156, and 0.069, respectively.

**Table 2:** Performance of different models in each cohort.

		AUC	Accuracy	Sensitivity	Specificity	PPV	NPV
**The training cohort**	The radiomics model	0.82 (0.76~0.90)	0.78 (0.70~0.84)	0.79 (0.69~0.86)	0.79 (0.62~0.91)	0.92 (0.83~0.96)	0.56 (0.41~0.70)
	The RECIST model	0.53 (0.44~0.62)	0.60 (0.51~0.68)	0.67 (0.57~0.76)	0.39 (0.24~0.57)	0.75 (0.64~0.83)	0.30 (0.18~0.45)
	The clinical model	0.69 (0.60~0.79)	0.62 (0.53~0.70)	0.55 (0.45~0.65)	0.81 (0.63~0.91)	0.89 (0.77~0.95)	0.40 (0.29~0.52)
	The combined model	0.83 (0.75~0.91)	0.81 (0.74~0.88)	0.88 (0.79~0.93)	0.64 (0.46~0.79)	0.87 (0.78~0.93)	0.66 (0.48~0.80)
**The internal validation cohort**	The radiomics model	0.77 (0.63~0.91)	0.73 (0.60~0.83)	0.74 (0.59~0.85)	0.69 (0.42~0.88)	0.88 (0.74~0.96)	0.46 (0.26~0.67)
	The RECIST model	0.67 (0.53~0.81)	0.73 (0.60~0.83)	0.78 (0.64~0.88)	0.56 (0.31~0.79)	0.85 (0.71~0.93)	0.45 (0.24~0.68)
	The clinical model	0.59 (0.43~0.74)	0.49 (0.36~0.61)	0.40 (0.27~0.55)	0.75 (0.47~0.92)	0.83 (0.62~0.95)	0.29 (0.16~0.45)
	The combined model	0.78 (0.65~0.92)	0.77 (0.65~0.87)	0.82 (0.68~0.91)	0.63 (0.34~0.84)	0.87 (0.74~0.95)	0.53 (0.30~0.75)
**The Dragon III cohort**	The radiomics model	0.78 (0.66~0.89)	0.70 (0.58~0.81)	0.54 (0.26 0.80)	0.74 (0.61~0.84)	0.32 (0.15~0.55)	0.88 (0.75~0.95)
	The clinical model	0.60 (0.46~0.74)	0.42 (0.31~0.55)	0.31 (0.10~0.61)	0.45 (0.32~0.58)	0.11 (0.04~0.27)	0.74 (0.56~0.87)
	The combined model	0.71 (0.59~0.84)	0.63 (0.51~0.75)	0.77 (0.46~0.94)	0.60 (0.47~0.73)	0.30 (0.16~0.49)	0.92 (0.78~0.98)
**The external validation cohort**	The radiomics model	0.72 (0.66~0.89)	0.49 (0.38~0.61)	0.91 (0.71~0.99)	0.31 (0.19~0.45)	0.37 (0.25~0.51)	0.89 (0.64~0.98)
	The clinical model	0.68 (0.56~0.81)	0.60 (0.48~0.71)	0.74 (0.51~0.89)	0.54 (0.40~0.68)	0.42 (0.27~0.58)	0.82 (0.65~0.93)
	The combined model	0.76 (0.64~0.87)	0.40 (0.29~0.52)	0.96 (0.76~0.99)	0.15 (0.07~0.29)	0.33 (0.23~0.46)	0.89 (0.51~0.99)
**The SOXA cohort**	The radiomics model	0.50 (0.27~0.73)	0.52 (0.32~0.71)	0.44 (0.21~0.69)	0.64 (0.32 0.88)	0.64 (0.32~0.88)	0.44 (0.21~0.69)
	The clinical model	0.57 (0.34~0.81)	0.56 (0.35~0.75)	0.63 (0.36~0.84)	0.46 (0.18~0.75)	0.63 (0.36~0.84)	0.46 (0.18~0.75)
	The combined model	0.51 (0.27~0.72)	0.59 (0.39~0.78)	0.75 (0.47~0.92)	0.36 (0.12~0.68)	0.63 (0.39~0.83)	0.50 (0.17~0.83)

AUC, area under the curve; NPV, negative predictive value; PPV, positive predictive value; RECIST, Response Evaluation Criteria in Solid Tumors. The SOXA cohort is defined as the patients receiving the regimen of apatinib in combination with SOX (S-1 and oxaliplatin).

### Model Comparison

The RECIST 1.1 showed poor performance to predict TRG, which reached AUCs of 0.53 (95% CI, 0.44~0.62) and 0.67 (95% CI, 0.53~0.81) in the training and internal validation cohorts, respectively. The radiomics model in the training cohort was significantly better than the RECIST model (p < 0.001), but no statistical difference was found in the internal validation cohort (p = 0.338). Detailed information on discriminative metrics for the RECIST models is presented in **Table 2**. The Kappa indices of the RECIST models of the training cohort and the internal validation cohort were 0.057 and 0.316, respectively.

The clinical model, incorporating age and tumor size in multivariate logistic regression, achieved AUCs of 0.69 (95% CI, 0.60~0.79) and 0.59 (95% CI, 0.43~0.74) in the training and internal validation cohorts for patients receiving the EOX regimen. For the Dragon III and external validation cohorts, the performance of the clinical models was 0.60 (95% CI, 0.46~0.74) and 0.68 (95% CI, 0.56~0.81), respectively. For patients receiving NAC regimens, the performance of the radiomics models was better than that of the clinical models to predict TRG in each cohort, although the marginal difference was only observed in the training cohort and the Dragon III cohort (p = 0.052 and p = 0.057, respectively). On the contrary, the clinical model showed a higher predictive value compared with the radiomics model in the SOXA cohort, with an AUC of 0.57 (95% CI, 0.34~0.81), and no statistical difference was found for the comparison (p = 0.664). Detailed information on discriminative metrics for the clinical models is presented in **Table 2**. The Kappa indices of the clinical models of the training cohort, the internal validation cohort, the Dragon III cohort, the external validation cohort, and the SOXA cohort were 0.269, 0.097, 0.145, 0.002, and 0.080, respectively.

**Figure 3:**
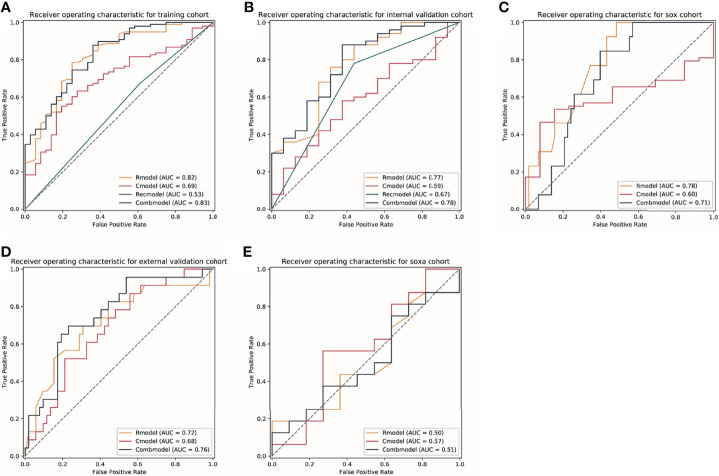
The receiver operator characteristic (ROC) curves for models in each cohort. **(A–E)** Represents ROC curves of models for patients in the training cohort, the internal validation cohort, the Dragon III cohort, the external validation cohort, and the SOXA cohort. Rmodel, the radiomics model; Cmodel, the clinical model; Recmodel, the RECIST model; Combmodel, the combined model. The SOX cohort is defined the patients receiving the regimen of S-1 and oxaliplatin; the SOXA cohort is defined as the patients receiving the regimen of apatinib in combination with SOX (S-1 and oxaliplatin). AUC, area under the curve.

The combined models that integrated the radiomics score and clinical risk factors showed limited improvement compared with the radiomics models in each cohort (**Table 2**). The results indicated a similar trend observed for the radiomics models, where a similar predictive power was found for patients receiving NAC regimens, while significantly declined performance was found in the SOXA regimen. When compared with the clinical models, the combined models in the training and internal validation cohorts presented statistically higher predictive value to TRG (p = 0.003 and p = 0.030, respectively). The ROC curves for all models in each cohort are presented in **Figure 3**. The Kappa indices of the combined models of the training cohort, the internal validation cohort, the Dragon III cohort, the external validation cohort, and the SOXA cohort were 0.521, 0.418, 0.073, 0.002, and 0.119, respectively.

**Figure 4:**
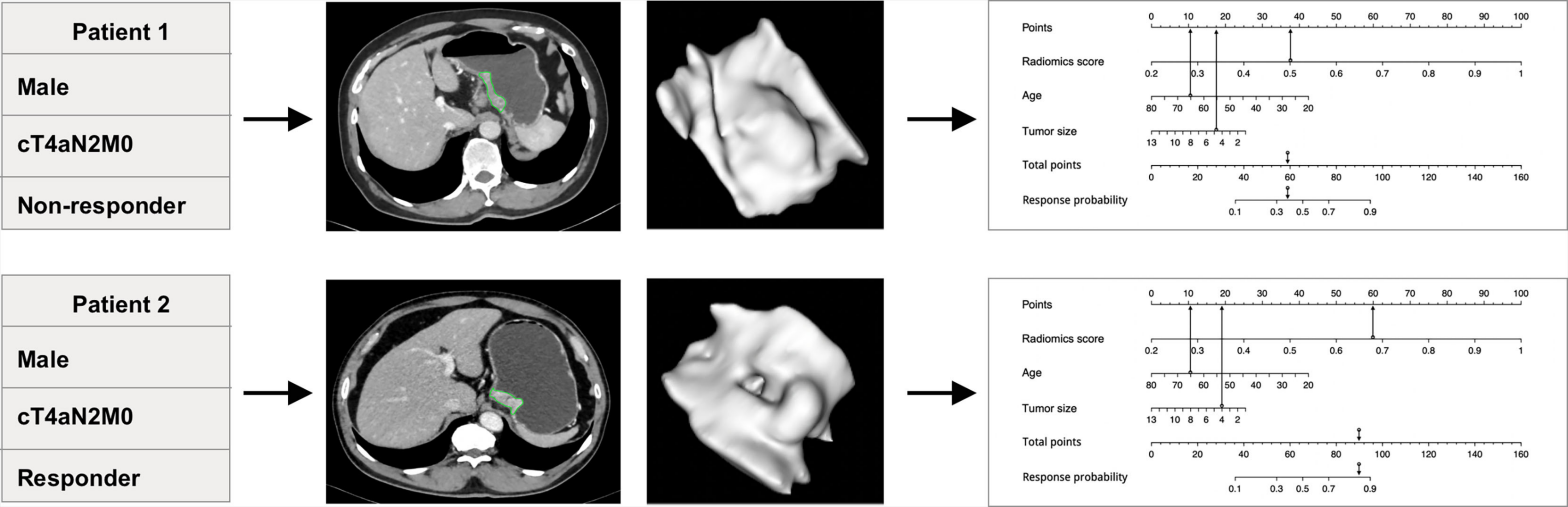
The predictive value of the individualized nomogram to predict tumor regression grade (TRG) for two patients receiving the EOX (epirubicin, oxaliplatin, and capecitabine) regimen. The two patients had similar clinical baseline information but different insensitivity to neoadjuvant chemotherapy treatment (both were men, 65 years old, cT4aN2M0, and similar tumor size). The individualized nomogram integrated radiomics score, age, and tumor size successfully predicted the outcomes of the patients, which mainly relied on the performance of radiomics score.

The nomogram indicated that individuals with lower radiomics scores, younger age, and smaller tumor size are prone to respond to NAC treatment; the calibration curve revealed that the radiomics model and the combined model showed better goodness of fit compared with the clinical model because lower Brier scores were observed (0.159 and 0.136 vs. 0.182) (**Supplementary Figure S2**). The DCA curve indicates that the combined model and the radiomics model have higher clinical net benefit than the clinical model (**Figure 4**, **Supplementary Figure S2**).

### Subgroup Analysis

We further analyzed the distribution difference of radiomics features in terms of response status between the SOX and FLOT regimens as well as the SOX and SOXA regimens using top 10 importance ranking features. In the Dragon III cohort, response rates for patients receiving the SOX and FLOT regimens were 18.5% and 18.2%, and there were five and eight responders in each regimen, respectively. In the non-responder group, no radiomics features showed a significant difference between the regimens (**Supplementary Figure S3**). However, in the responder group, four radiomics features were statistically different between the two regimens (**Supplementary Figure S4**).

The response rate for patients receiving the SOXA regimen was 59.3% (16/27). In the non-responder group, 3 of 10 radiomics features showed a significant difference, and they were all from the delayed phase images (**Supplementary Figure S5**). Intriguingly, those features also presented significance between the two regimens in the responder group (**Supplementary Figure S6**). Apart from that, another radiomics features also shared a significantly different distribution.

## Discussion

In this study, we developed and validated an AI method using quantitative imaging named radiomics and successfully predicted TRG for AGC patients treated with NAC, which showed generalized power among different NAC regimens by validating its predictive value in the Dragon III cohort and an external validation cohort. More importantly, radiomics noninvasively revealed that NAC regimens and molecular targeted therapy probably shared different pharmacological effects on tumors. This observation was identified by a significantly declined performance of the radiomics model in the SOXA cohort and the results from the subgroup analysis. Our findings are helpful to evaluate the benefit of NAC before treatment and provide the opportunity to timely adjust to an appropriate strategy for patients insensitive to NAC.

Previous studies have demonstrated the predictive value of radiomics in evaluating pathological response after NAC for GC (21, 31, 32). However, few pieces of research reported the generalization of radiomics among different regimens except for a study in non–small cell lung cancer, which has first demonstrated that radiomics could serve as an early indication for different systemic anticancer therapies (19). To verify whether radiomics could exert predictive value to NAC for GC, we first trained and validated our radiomics model in patients treated with the EOX regimen. The radiomics model constructed by selected 28 radiomics features achieved a comparable AUC (0.77) in the internal validation cohort compared with previously reported performance from 0.722 to 0.82 (21, 31, 32). To investigate the generalization of our radiomics model in neoadjuvant agents, we further applied it on the Dragon III cohort and external validation cohort including five different NAC regimens. The radiomics model continued to show its efficacy in the two cohorts, identifying the generalization of our model. For comparison, the commonly used RECIST 1.1 criteria only showed limited value to predict tumor response to NAC, urging the application of our radiomics model as an early prediction tool for AGC after neoadjuvant treatment. Overall, radiomics shows its potentiality in early prediction of tumor response for GC patients receiving NAC, and more importantly, this efficacy is generalizable among different NAC regimens.

The FLOT regimen is a new candidate NAC approach for AGC patients. Intriguingly, in the Dragon III study, no significantly improved response rate was observed between the FLOT and SOX regimens (23). We also performed a subgroup analysis between the two regimens to investigate whether the radiomics features could provide evidence for the finding in the clinical trial. No features were found statistically different in the non-responders between the two groups, which was consistent with what was observed in the Dragon III study. However, four radiomics features demonstrated a significant difference in the responders between the two groups. Due to the low response rate observed for the two regimens (18.2% and 18.5% for FLOT and SOX, respectively), the sample size in the responder subgroup for each regimen was small, which probably weakened the efficacy of the test. Therefore, whether there was any tumor heterogeneity for patients who were sensitive to the regimens remained to be elucidated.

Unlike indistinctive cytotoxicity from conventional chemotherapy drugs, apatinib was an antiangiogenic agent, which highly selectively targets vascular endothelial growth factor receptor 2 (VEGFR-2) tyrosine kinase, mediating the growth of the tumor by inhibiting angiogenesis (33). Apatinib alone exerts its antitumor effects by inhibiting tumor growth, reducing microvascular density, and enhancing apoptosis (34). This molecular targeted drug was considered as third- or subsequent-line therapy for chemotherapy-refractory advanced or metastatic GC in phase II and III studies, with good tolerance and improved survival (35, 36). The synergistic antineoplastic effects of apatinib combined with chemotherapy agents were observed in preclinical studies (37, 38), which was demonstrated in the clinical practice setting, in combination with preoperative SOX regimen to improve the pathological response of AGC (24). However, the intrinsic changes in tumor heterogeneity initiated by apatinib have not been clarified. In this study, the radiomics model constructed by the NAC regimen performed poorly in the SOXA cohort, revealing the existence of the underlying difference between the two different neoadjuvant approaches. We then performed a subgroup analysis between patients receiving the SOX regimen and the SOXA regimen to investigate the added value of apatinib. Due to the SOXA cohort being a single-arm clinical trial, patients receiving the SOX regimens were from the Dragon III study. Three key radiomics features simultaneously expressed differences in both the responder and non-responder groups, indicating that the discriminative tumor heterogeneity after the addition of apatinib could be detected early by quantitative imaging. Meanwhile, the clinical model maintained a similar performance regardless of which neoadjuvant approach was taken, laterally highlighting the importance of our radiomics model for a discriminative capability for NAC and molecular targeted therapy. Those findings suggested that our radiomics model is helpful to screen patients insensitive to NAC regimens before treatment and thus provide the opportunity for those patients to choose appropriate treatment such as molecular targeted therapy or immunotherapy.

Our study achieved an RQS of 30 (83.3% of ideal score), which is higher than most radiomics studies (reviews report an average RQS percentage ranging from 9.4% to 26.1%) (39–42), guaranteeing the robustness and reproducibility of our study. The calibration curve showed well goodness of fit of the radiomics model, indicating the robustness of the results. The DCA curve revealed that our radiomics model presented higher net benefit in almost the whole period compared with the clinical model, increasing the clinical use of radiomics in providing advice for patients if they should receive NAC treatment.

Our study has several limitations. Except for data from two clinical trials, patients receiving the EOX regimen in the external validation cohort were retrospectively recruited, and bias selection was inevitable in this proof-of-concept study. Also, a potential confounder is that we had limited information in molecular or genetic aspects for those patients, and the diversity among patients may lead to different responses to neoadjuvant therapies. The response rate in each cohort varied, which conformed to the complex condition in the real world. Furthermore, except for the EOX regimen, limited sample size in other NAC regimens hindered the construction of models for each cohort or NAC regimen to investigate the similarity of radiomics features expressed in different NAC regimens. For the same reason, an independent radiomics model for the SOXA cohort was also not realized. Therefore, we could conclude that radiomics was capable of predicting different pharmacological effects on tumors between NAC regimens and molecular targeted therapy, but the nuances that reflected radiomics features between the two different neoadjuvant approaches remained to be further elucidated. Although TRG is reported as a surrogate for prognosis, the predictive value of our radiomics model needs to be elucidated in further investigation with follow-up evidence.

In conclusion, in this study, we demonstrated that our radiomics model is generalizable to predict TRG for AGC patients receiving NAC treatments, which is beneficial to transform appropriate treatment for GC, especially for those insensitive to NAC. We also identified the intrinsic difference between cytotoxic neoadjuvant chemo agents and molecular targeted therapy from a radiomics perspective, which is helpful to construct individualized models for different neoadjuvant approaches.

## Data Availability Statement

The original contributions presented in the study are included in the article/**Supplementary Material**. Further inquiries can be directed to the corresponding authors.

## Ethics Statement

The studies involving human participants were reviewed and approved by the ethics committee of Ruijin Hospital and Peking University Cancer Hospital and Institute. The ethics committee waived the requirement of written informed consent for participation. Written informed consent was obtained from the individual(s) for the publication of any potentially identifiable images or data included in this article.

## Author Contributions

CL, ZZ and QM contributed to the conception of the study and design of the study. YC, WX and Y-LL performed the data collection and validation. YC wrote the first draft of the manuscript. All authors contributed to manuscript revision, read, and approved the submitted version.

## Funding

This work was funded by Shanghai Science and Technology Commission Science and Technology Innovation Action Clinical Innovation Field (18411953000), the National Natural Science Foundation of China (81771789), and Beijing Natural Science Foundation (Z180001).

## Conflict of Interest

ZX is employed by Siemens Healthineers Ltd. MW is employed by Siemens Healthcare GmbH.

The remaining authors declare that the research was conducted in the absence of any commercial or financial relationships that could be construed as a potential conflict of interest.

## Publisher’s Note

All claims expressed in this article are solely those of the authors and do not necessarily represent those of their affiliated organizations, or those of the publisher, the editors and the reviewers. Any product that may be evaluated in this article, or claim that may be made by its manufacturer, is not guaranteed or endorsed by the publisher.

## Abbreviations

AGC, advanced gastric cancer; AI, artificial intelligence; AUC, area under the curve; CA19-9, carbohydrate antigen 19-9; CEA, carcinoembryonic antigen; CECT, contrast-enhanced computed tomography; GC, gastric cancer; IBSI, image biomarker standardization initiative; ICC, intraclass correlation coefficient; NAC, neoadjuvant chemotherapy; NPV, negative predictive value; PPV, positive predictive value; RECIST, Response Evaluation Criteria in Solid Tumors; RF, random forest; ROC, receiver operating characteristic; RQS, radiomics quality score; TRG, tumor regression grade; VOI, volume of interest.
