# Supplementary material for: CT-Based Radiomics Showing Generalization to Predict Tumor Regression Grade for Advanced Gastric Cancer Treated With Neoadjuvant Chemotherapy
Source: Front Oncol. 2022 Feb 25;12:758863. doi: 10.3389/fonc.2022.758863 (PMC8913538; doi:10.3389/fonc.2022.758863)
Supplement: Supplementary file 1 [file DataSheet_1.docx]

Supplementary Material

# Supplementary S1

Tumor invasion and lymph node status were evaluated according to cTNM stage based on baseline CECT images. Tumor location was classified as upper, middle, lower, and diffuse-type if the tumor stretched over two sites. Differentiation status was classified as good, moderate, or poor status for adenocarcinoma according to pathological evaluation by biopsy. If more than 50% of viable tumor cells were signet-ring cells, then signet-ring cell carcinoma (SRCC) was recorded; if biopsy tissues were diagnosed as adenocarcinoma but unable to accurately assess the differentiation status, then “not evaluated” was recorded. The normal upper level of CEA and CA199 was 5ng/ml and 35u/ml, respectively.

# Supplementary S2

For patients receiving the EOX regimen, epirubicin (50 mg/m2) and oxaliplatin (132 mg/m2) were administered intravenously on 1st day and capecitabine was administered orally twice daily at a dose of 625 mg/m2 on days 1st-14th. This regimen was repeated every 21 days and repeated for 3 preoperative cycles.

For patients receiving the SOX regimen in the external validation cohort, S-1 (80 mg/m2) was administrated orally twice daily on days 1st−14th and oxaliplatin (130 mg/m2) was administrated intravenously on day one for each cycle. This regimen was repeated for three preoperative cycles. For the XELOX regimen, oxaliplatin (130 mg/m2) was administrated intravenously on day 1st, and capecitabine (1000 mg/m2) was administrated orally twice daily on days 1st–14th. This regimen was repeated every 21 days and repeated for 3 preoperative cycles. For the FOLFOX regimen, oxaliplatin (100 mg/m2) and folinic acid (400 mg/m2) were administered intravenously for two hours on day 1st, followed by 5‐FU (2400 mg/m2) by continuous infusion for 46 hours. This regimen was repeated every 14 days and repeated for 2-4 cycles.

# Supplementary S3

A total of 110 original radiomics features (without any pre-processing producers applied) were extracted from each phase, including first-order features (n = 18), texture features (n = 75) and shape features (n = 17). The texture radiomics features consisted of five subgroups of features, including gray level co-occurrence matrix (GLCM, n = 24), gray-level difference method (GLDM, n = 14), grey level run length matrix (GLRLM, n = 16), grey level size zone matrix (GLSZM, n = 16) and neighborhood grey tone difference matrix (NGTDM, n = 5). We applied wavelet transformation for images and four types of mathematical transformations (including square, square root, exponential and logarithm) for features and then extracted corresponding radiomics features after those steps. Pre-processing producers included wavelet transformation for images and four types of mathematical transformations (including square, square root, exponential and logarithm) for features. For wavelet transformation, both of high-filter (H) and low-filter (L) were applied in x, y, z dimensions; therefore, eight subgroups of wavelet transformation were generated (HHH, HHL, HLH, HLL, LHH, LHL, LLH and LLL). Due to resisted nature of voxel for shape features, only first-order and texture radiomics features were extracted after pre-processing producers. Therefore, for each feature in the first-order and texture categories, 12 new radiomics features were generated and extracted. Therefore, there were 1116 [12 × (18 + 75)] new radiomics feature extracted after pre-processing producers. Combined with the 110 original radiomics features, there was a total of 1226 radiomics features extracted from each image series.

**
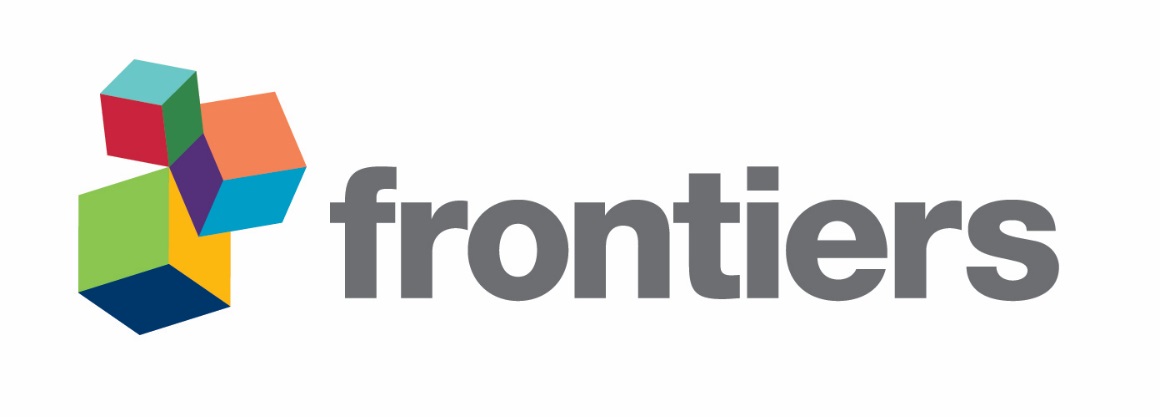
**

Supplementary Table S1 Information on CT imaging protocol

| **Manufacturers Model Name** | **Tube current (mAs)** | **Tube volume (kVp)** | **Pitch (mm)** | **Matrix** | **Slice thickness (mm)** |
| --- | --- | --- | --- | --- | --- |
| Canon Aquilion ONE | 100 | 100 | 0.828 | 512×512 | 5 |
| GE Discovery CT750 | 300 | 120 | 0.984 | 512×512 | 1.5 |
| GE Revolution | 103 | 120 | 0.758 | 512×512 | 5 |
| Philips IQon - Spectral CT | 108 | 120 | 0.765 | 512×512 | 5 |
| Siemens SOMATOM  Force | 280 | 120 | 0.6 | 512×512 | 1.5 |
| Siemens SOMATOM  Definition Flash | 360 | 120 | 0.6 | 512×512 | 1.5 |

Supplementary Table S2 Package resources used in this study

| **Statistical analysis** | **Packages** | **Version** |
| --- | --- | --- |
| Stability test: intraclass correlation coefficient | ‘irr’ in R | 0.84.1 |
| Clinical and combination model establishment: multivariate logistic regression | ‘rms’ in R | 6.0-1 |
| Model performance evaluation: Decision Curve Analysis | ‘rmda’ in R | 1.6 |
| Model performance evaluation: Delong Test and 95%CI for ROC curve analysis | ‘pROC’ in R | 1.16.2 |
| Nomogram generation | ‘rms’ in R | 6.0-1 |
| Model establishment and radiomics score generation: Random Forest | ‘scikit-learn’ in Python | 0.13.2 |
| Parameter tunning: Grid Search with Cross validation | ‘scikit-learn’ in Python | 0.13.2 |
| Model performance evaluation: Calibration curve and Brier score calculation | ‘scikit-learn’ in Python | 0.13.2 |
| Feature selection: Mutual information calculation | ‘scikit-learn’ in Python | 0.13.2 |

Supplementary Table S3 Statistics for Baseline information between responders and non-responders on patients in the training cohort

|  | The training cohort | | p value |
| --- | --- | --- | --- |
|  | Responder  (n = 98) | Non-responder  (n = 36) |  |
| **Age** | 57.5 ± 10.9 | 62.0 ± 7.0 | 0.023 |
| **Gender (female, %)** | 29 (29.6%) | 12 (33.3%) | 0.837 |
| **Tumor invasion** |  |  | 1 |
| cT4 | 98 (100.0%) | 36 (100.0%) |  |
| **Lymph node status** |  |  | 0.529 |
| cN1 | 19 (19.4%) | 4 (11.1%) |  |
| cN2 | 39 (39.8%) | 16 (44.4%) |  |
| cN3 | 40 (40.8%) | 16 (44.4%) |  |
| **Tumor size (cm)** | 6.18 (2.35) | 7.22 (2.07) | 0.020 |
| **Tumor location** |  |  | 0.132 |
| upper | 19 (19.4%) | 14 (38.9%) |  |
| middle | 29 (29.6%) | 9 (25%) |  |
| lower | 32 (32.7%) | 9 (25%) |  |
| diffuse | 18 (18.4%) | 4 (11.1%) |  |
| **CA19-9 (positive, %)** | 33 (24.6%) | 10 (27.8%) | 0.774 |
| **CEA (positive, %)** | 27 (27.6%) | 12 (33.3%) | 0.661 |

Abbreviations: CA19-9: carbohydrate antigen 19-9; CEA: carcinoembryonic antigen.

Supplementary Table S4 Radiomics quality score of our study

| Criterion |  | Comments | Points |
| --- | --- | --- | --- |
| 1 | Image protocol quality | The image protocol used in this study was well-documented. | 2 |
| 2 | Multiple segmentations | Segmentation was performed by two radiologists and reliability analysis was evaluated by intra-class coefficient. | 1 |
| 3 | Phantom study on all scanners | NA | 0 |
| 4 | Imaging at multiple time points | NA. | 0 |
| 5 | Feature reduction or adjustment for multiple testing | Features reduction was completed by mutual information and random forest. | 3 |
| 6 | Multivariable analysis with non-radiomics features | The radiomics models was correlated with clinical factors and a radiomics-clinical combined model was constructed. | 1 |
| 7 | Detect and discuss biological correlates | We used the radiomics model to predict tumor regression grade in the SOXA cohort and discussed the difference the SOX and SOXA regimens after apatinib added. | 1 |
| 8 | Cut-off analyses | NA | 0 |
| 9 | Discrimination statistics | Discriminative metrics included AUC, accuracy, sensitivity, specificity, PPV, and NPV. Five cross-validation was used when tuning with random forest in the training cohort. | 2 |
| 10 | Calibration statistics | Calibration curves was performed to observe the goodness of fit of our models. Resampling was used for the calibration of nomogram. | 2 |
| 11 | Prospective study registered in a trial database | Data from two clinical trials (ClinicalTrials.gov: NCT03636893 and ChiCTR.gov.cn: ChiCTR-OPC-16010061) were included. | 7 |
| 12 | Validation | The performance of radiomics was validated in a testing cohort, an internal validation cohort and an external validation cohort. | 3 |
| 13 | Comparison to ‘gold standard’ | It lacks of gold standard to predict outcomes of gastric cancer after NAC. Instead, we used a clinical model for comparison. | 2 |
| 14 | Potential clinical utility | A nomogram and a decision curve analysis were implemented for each survival arm to investigate the potential clinical unity of our model. | 2 |
| 15 | Cost-effectiveness analysis | NA | 0 |
| 16 | Open science and data | The scans used was well-documented. Radiomics features were available in the pyradiomics library and compliant with the benchmarks of the image biomarker standardization initiative ( <https://ibsi.readthedocs.io/en/latest/index.html>). The ROIs delineated were representative for the primary tumor slice by slice. The code and ROIs used in this study would be open source on reasonable requirement. | 4 |
| **Total points** | | **30/36 (83.3%)** | |

Abbreviation: ROC: receiver operating characteristic.

**
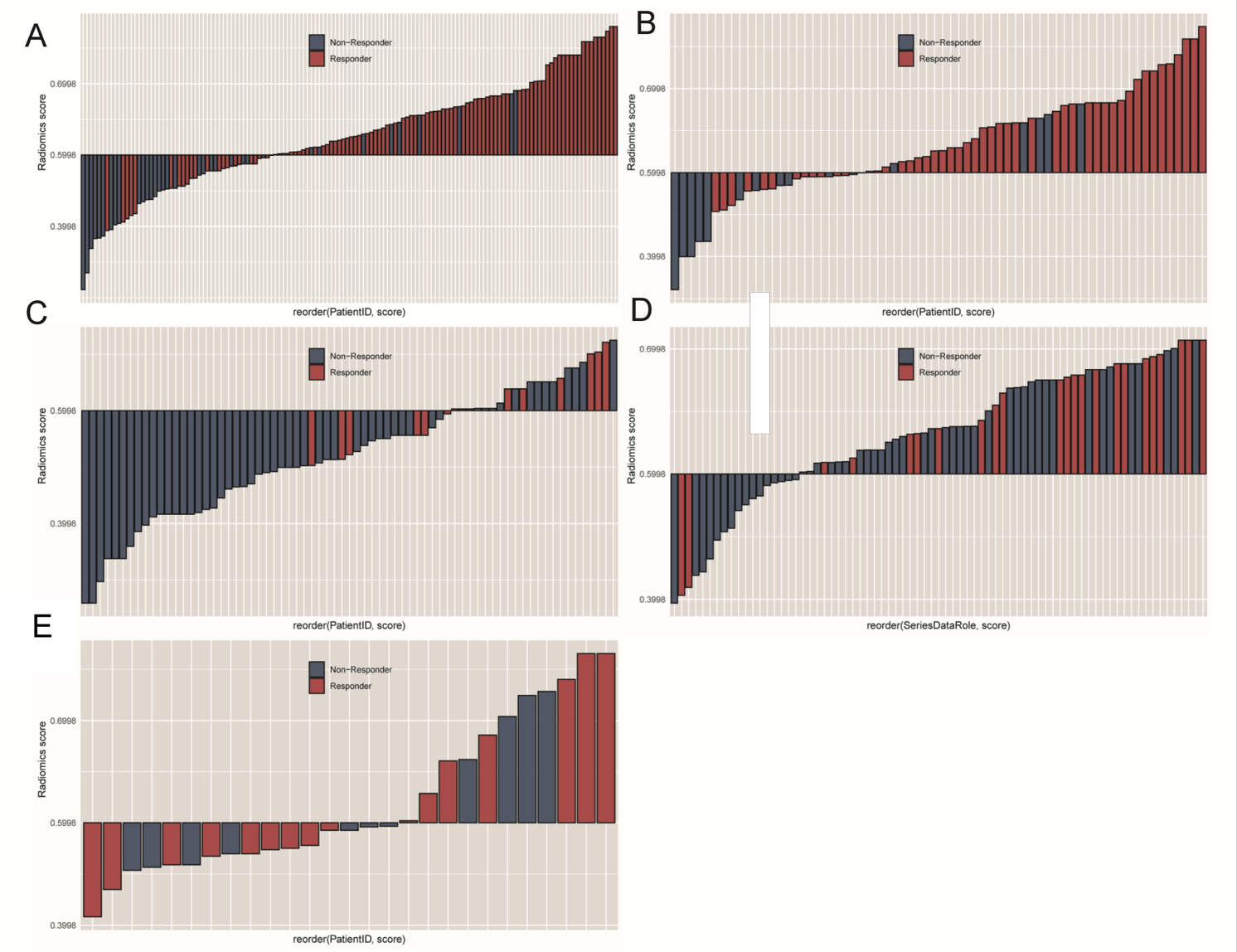
**

Supplementary Figure S1 Radiomics score for patients in each cohort

(A-E) represents radiomics score for patients in the training cohort, the internal validation cohort, the Dragon III cohort, the external validation cohort, and the SOXA cohort. For each cohort, responders were prone to have higher radiomics scores than non-responders. The SOXA cohort is defined as the patients receiving the regimen of apatinib in combination with SOX (S-1 and oxaliplatin).


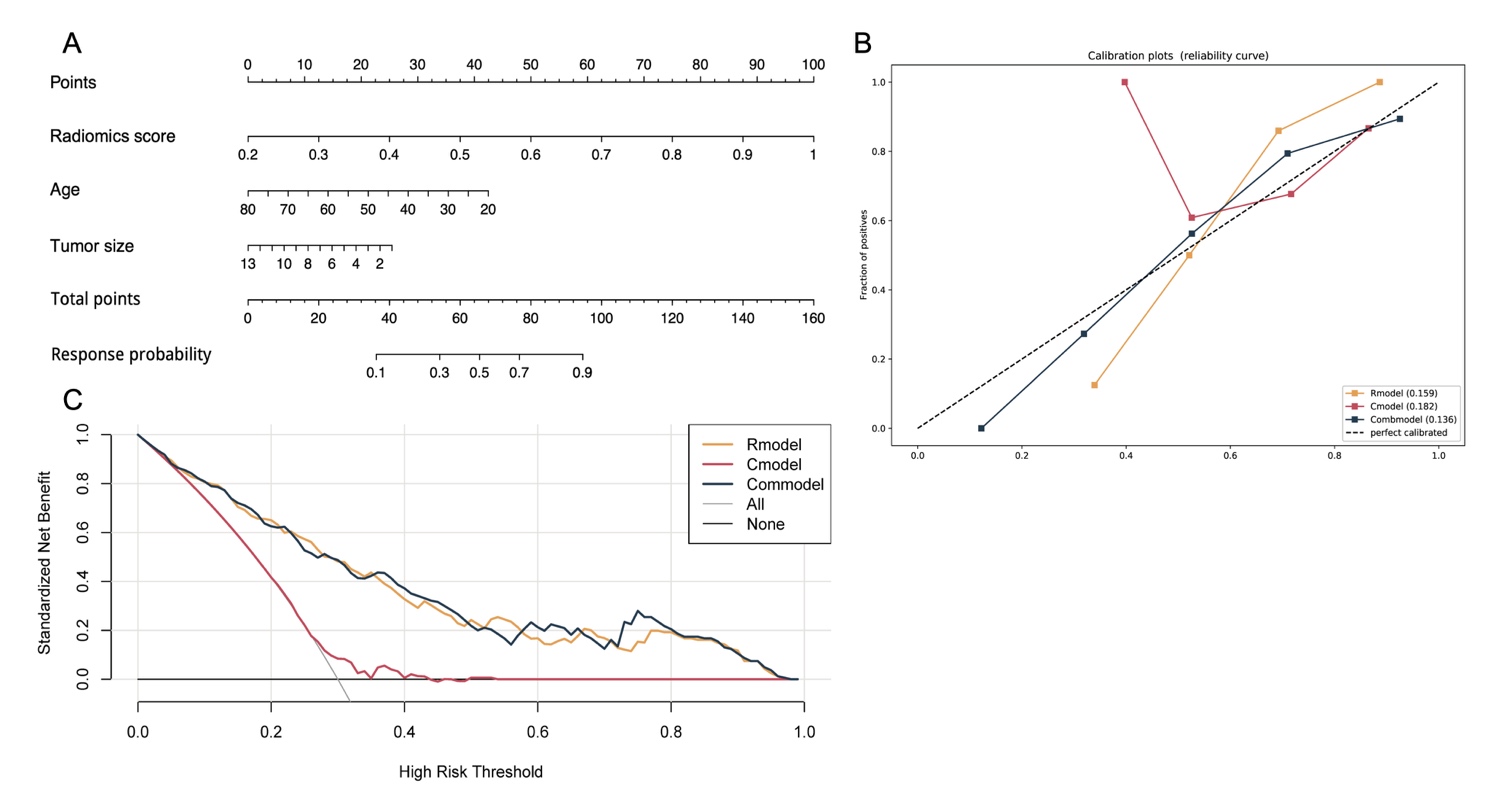


Supplementary Figure S2 The clinical use of our radiomics model in the training cohort

(A) shows nomogram for individuals treated with the EOX regimen in the training cohort; (B) shows the goodness of fit of the predicted radiomics model (Rmodel), clinical model (Cmodel), and the combined model (Combmodel) compared with what happened (the perfected calibrated line). The radiomics model and the combined model both showed better goodness of fit compared with the clinical model, with lower Brier scores. (C) shows the clinical net benefits for patients to predict response probabilities by the radiomics model, the clinical model, and the combined model using decision curve analysis. The radiomics model and the combined model showed similar performance, which both had a higher net benefit than the clinical model in almost the whole period. The EOX regimen is epirubicin, oxaliplatin, and capecitabine.


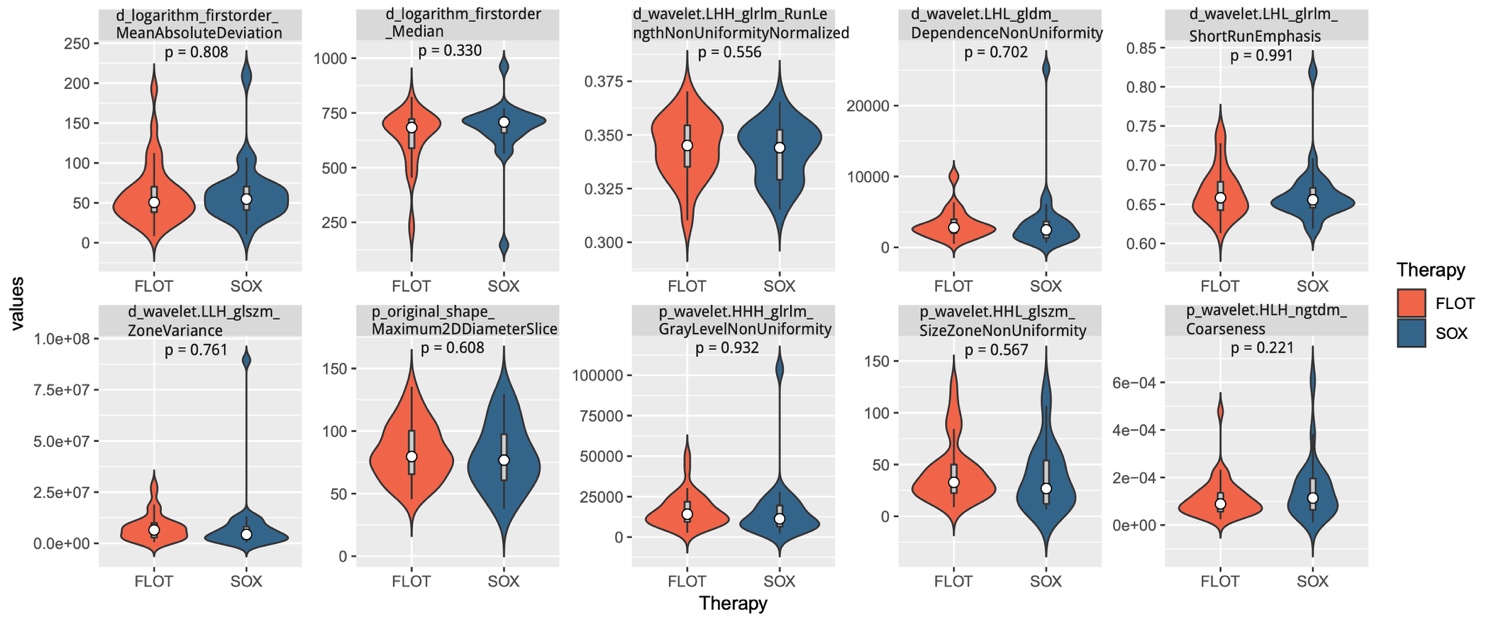


Supplementary Figure S3 Subgroup analysis of 10 radiomics features for patients receiving the SOX and FLOT regimens in the non-responder group

No features indicated statistically different. The feature name is ordered by the following rule: phase (p or d represents the venous-portal phase or the delayed phase) _ pre-processing producer_feature category_feature name. For example, for the first feature, i.e. d_logarithm_firstorder_MeanAbsoluteDeviation indicates the feature is named as MeanAbsoluteDeviation from the firstorder category, with transformation by logarithm. For the regimens, FLOT (5-fluorouracil, leucovorin, docetaxel and oxaliplatin), SOX (S-1 and oxaliplatin).


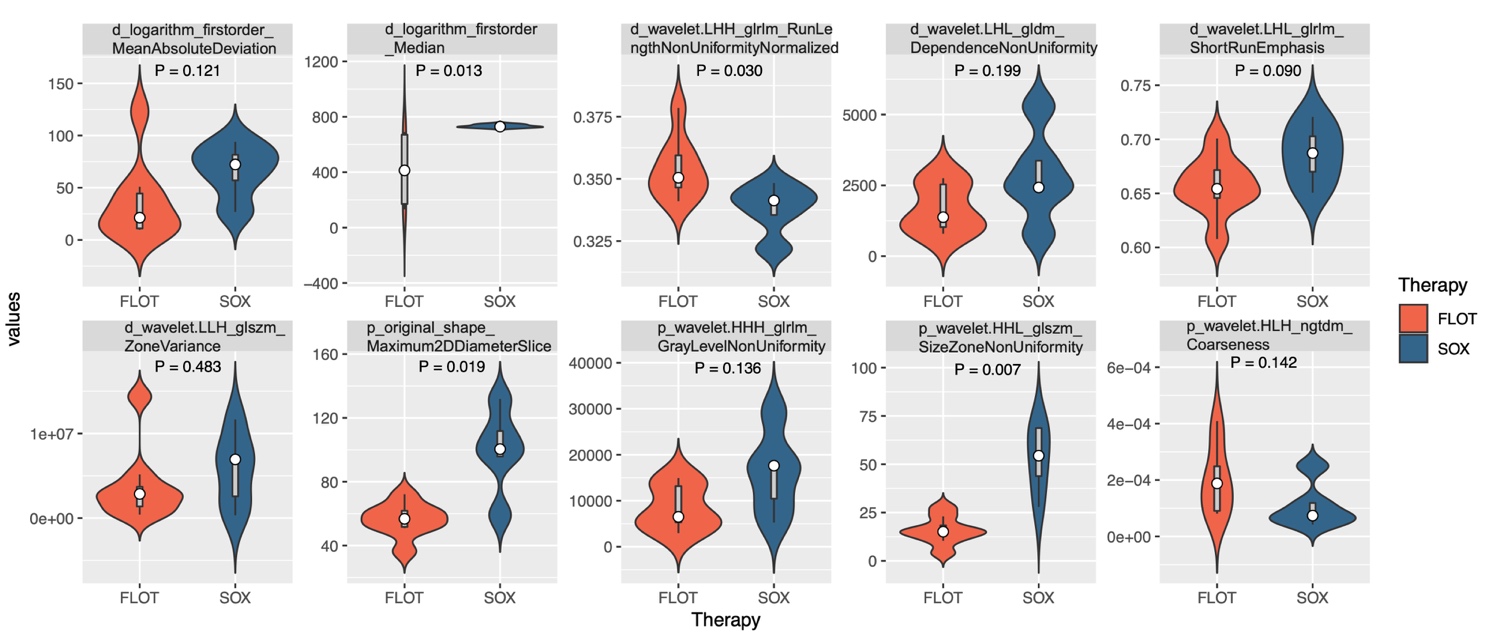


Supplementary Figure S4 Subgroup analysis of 10 radiomics features for patients receiving the SOX and FLOT regimens in the responder group

Four radiomics features showed statistically different. The feature name is ordered aforementioned. For the regimens, FLOT (5-fluorouracil, leucovorin, docetaxel and oxaliplatin), SOX (S-1 and oxaliplatin).


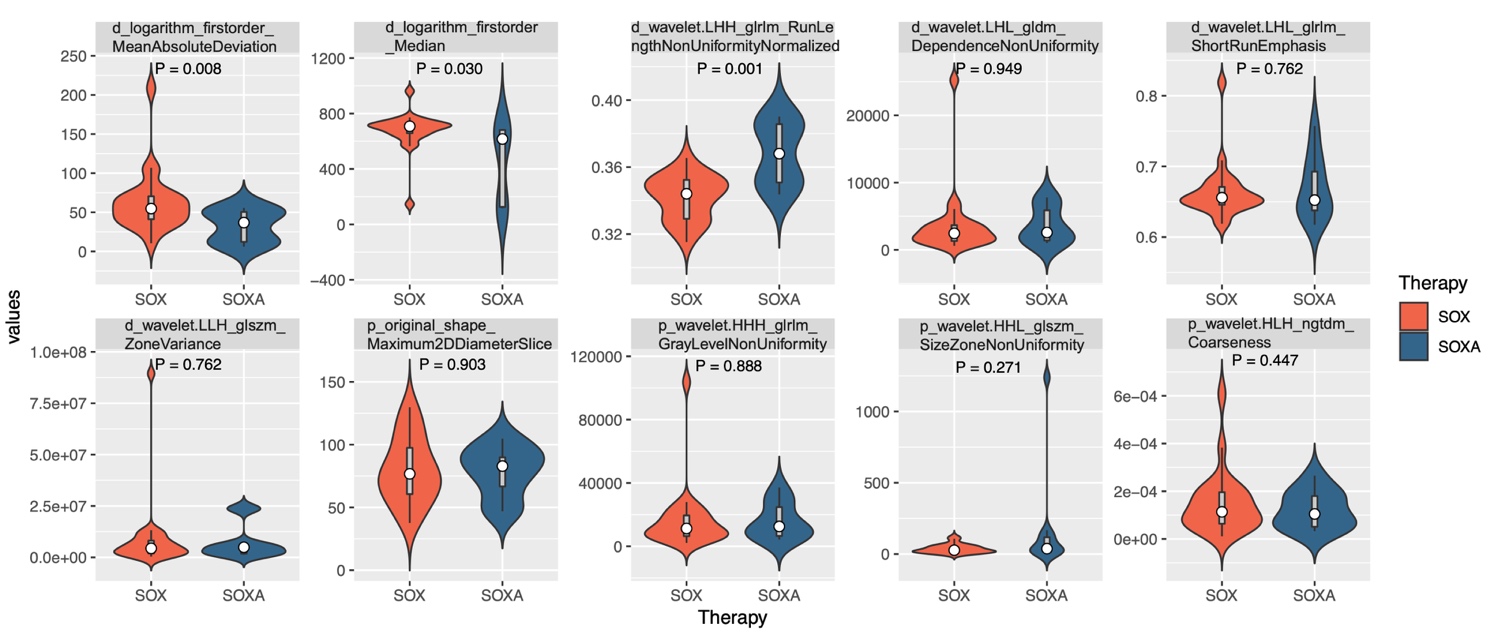


Supplementary Figure S5 Subgroup analysis of 10 radiomics features for patients receiving the SOX and SOXA regimens in the non-responder group

Three radiomics features showed statistically different. The feature name is ordered aforementioned. For the regimens, SOX (S-1 and oxaliplatin), SOXA (apatinib in combination with SOX).


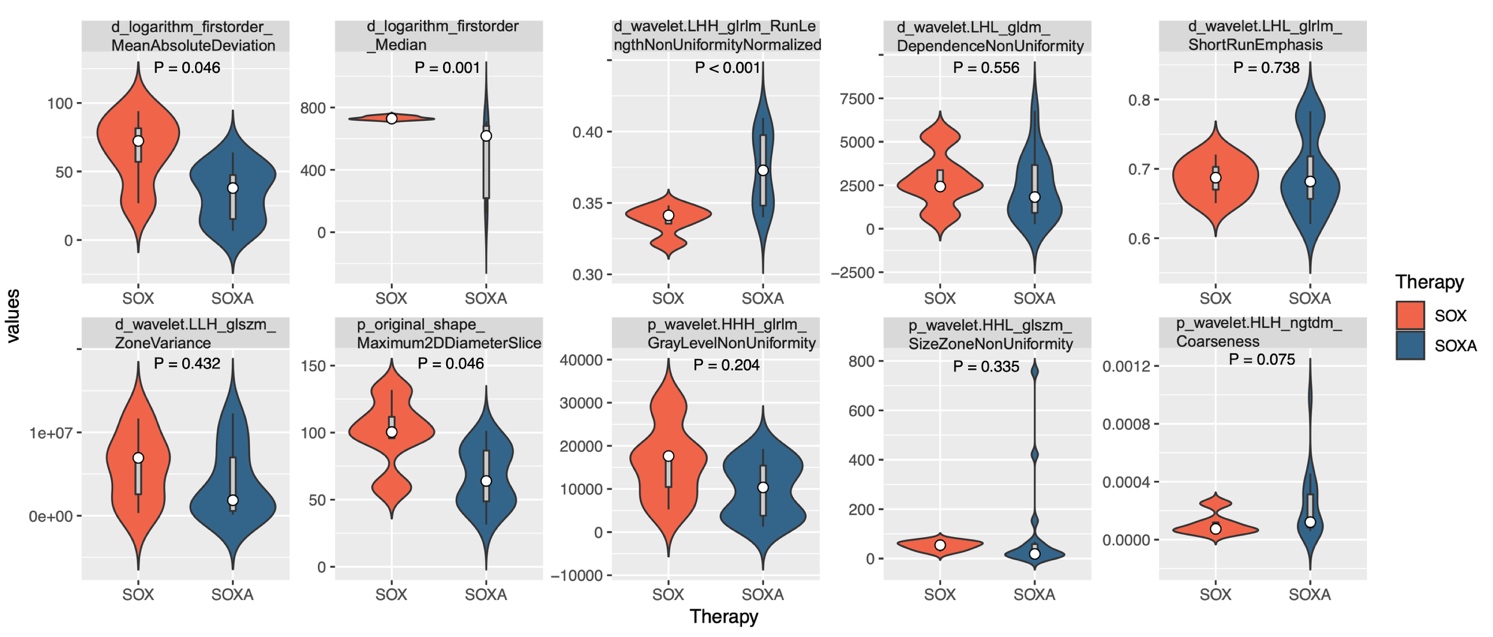


Supplementary Figure S6 Subgroup analysis of 10 radiomics features for patients receiving the SOX and SOXA regimens in the responder group

Four features showed statistically different. The feature name is ordered aforementioned. For the regimens, SOX (S-1 and oxaliplatin), SOXA (apatinib in combination with SOX).
